# Insights into the recognition of hypermucoviscous *Klebsiella pneumoniae* clinical isolates by innate immune lectins of the Siglec and galectin families

**DOI:** 10.3389/fimmu.2024.1436039

**Published:** 2024-08-01

**Authors:** María Asunción Campanero-Rhodes, Sara Martí, Noelia Hernández-Ortiz, Meritxell Cubero, June Ereño-Orbea, Ana Ardá, Jesús Jiménez-Barbero, Carmen Ardanuy, Dolores Solís

**Affiliations:** ^1^ Department of Biological Physical Chemistry, Instituto de Química Física Blas Cabrera, Consejo Superior de Investigaciones Científicas, Madrid, Spain; ^2^ Centro de Investigación Biomédica en Red de Enfermedades Respiratorias (CIBERES), Instituto de Salud Carlos III, Madrid, Spain; ^3^ Microbiology Department, Hospital Universitari Bellvitge, University of Barcelona-Fundación Instituto de Investigación Biomédica de Bellvitge, L’Hospitalet de Llobregat, Spain; ^4^ CIC bioGUNE - Center for Cooperative Research in Biosciences, Basque Research & Technology Alliance (BRTA), Bizkaia Technology Park, Derio, Spain; ^5^ Ikerbasque, Basque Foundation for Science, Bilbao, Spain; ^6^ Department of Organic Chemistry, II Faculty of Science and Technology University of the Basque Country, EHU/UPV, Leioa, Spain; ^7^ Department of Pathology and Experimental Therapeutics, University of Barcelona, Barcelona, Spain

**Keywords:** *Klebsiella pneumoniae*, hypermucoviscosity, bacterial cell surface carbohydrates, lectins, Siglecs, galectins

## Abstract

*Klebsiella pneumoniae* is an opportunistic bacterium that frequently colonizes the nasopharynx and gastrointestinal tract and can also cause severe infections when invading other tissues, particularly in immunocompromised individuals. Moreover, *K. pneumoniae* variants exhibiting a hypermucoviscous (HMV) phenotype are usually associated with hypervirulent strains that can produce invasive infections even in immunocompetent individuals. Major carbohydrate structures displayed on the *K. pneumoniae* surface are the polysaccharide capsule and the lipopolysaccharide, which presents an O-polysaccharide chain in its outermost part. Various capsular and O-chain structures have been described. Of note, production of a thick capsule is frequently observed in HMV variants. Here we examined the surface sugar epitopes of a collection of HMV and non-HMV *K. pneumoniae* clinical isolates and their recognition by several Siglecs and galectins, two lectin families of the innate immune system, using bacteria microarrays as main tool. No significant differences among isolates in sialic acid content or recognition by Siglecs were observed. In contrast, analysis of the binding of model lectins with diverse carbohydrate-binding specificities revealed striking differences in the recognition by galactose- and mannose-specific lectins, which correlated with the binding or lack of binding of galectins and pointed to the O-chain as the plausible ligand. Fluorescence microscopy and microarray analyses of galectin-9 binding to entire cells and outer membranes of two representative HMV isolates supported the bacteria microarray results. In addition, Western blot analysis of the binding of galectin-9 to outer membranes unveiled protein bands recognized by this galectin, and fingerprint analysis of these bands identified several proteins containing potential *O*-glycosylation sites, thus broadening the spectrum of possible galectin ligands on the *K. pneumoniae* surface. Moreover, Siglecs and galectins apparently target different structures on *K. pneumoniae* surfaces, thereby behaving as non-redundant complementary tools of the innate immune system.

## 1 Introduction

Bacterial cells are frequently covered by a polysaccharide capsule that plays an important role in virulence (1, 2). The capsule may promote adherence to host cells and also shields inner bacterial surface structures from recognition by the host immune system, preventing complement activation and phagocytosis. In fact, bacterial virulence is commonly attenuated in the absence of the capsule. Conversely, many pathogenic bacteria boost their encapsulation when present in niches containing abundant host immune factors. Production of a thick polysaccharide capsule is a recurrent trait of *Klebsiella pneumoniae* cells exhibiting a hypermucoviscous (HMV) phenotype (3–5).

*K. pneumoniae* is an opportunistic Gram-negative bacterium that frequently colonizes the nasopharynx and gastrointestinal tract. Up to 79 *K. pneumoniae* capsular (K antigen) serotypes have been identified to date (3, 6) of which K1 and K2 serotypes are associated with higher virulence. “Classical” *K. pneumoniae* variants can cause a range of infections, including pneumonia, meningitis, bloodstream and urinary tract infection, particularly when infecting immunocompromised individuals, while HMV *K. pneumoniae* can produce invasive infections even in healthy, immunocompetent individuals (3–5). In these variants, hypercapsule production is frequently associated with the presence of the chromosomally encoded hypermucoviscosity gene A (*mag*A) and/or the plasmid gene regulator of the mucoid phenotype A (*rmp*A) (7). The *magA* gene is specific of serotype K1 and codes for the outer membrane protein wzi, which has been found to be involved in surface assembly of the capsule in *Escherichia coli* (8). The *wzy* gene is conserved in all capsular types of *K. pneumoniae* (6), so *magA* has been renamed as *wzy_K1*. On the other hand, the *rmp*A gene encodes a transcriptional regulator of the *rmpC* and *rmpD* genes, which are independently involved in capsule production and in regulation of capsule chain length and HMV, respectively (9–11). Other genes, as *kvrA, kvrB*, and *rcsB*, have been reported to regulate *rmpA* expression and capsule production (4, 9, 12). Furthermore, the HMV phenotype has also been detected in *mag*A-/*rmp*A- isolates (13). Recently, the emergence of hypervirulent *K. pneumoniae* strains carrying carbapenemase genes has been described in some European countries, being a cause of concern and deserving a surveillance program (14).

*K. pneumoniae* capsular polysaccharides contain uronic acids that confer the capsule an acidic character, important for bacterial survival. The presence of negatively charged sialic acid residues has also been detected in some strains (15–18). Cell surface display of sialic acid is a molecular mimicry mechanism exploited by several bacteria to subvert or even exploit the host immune system (19). Interestingly, a significantly higher concentration of sialic acid in capsular extracts of the HMV *K. pneumoniae* isolate KP-M1 compared to non-HMV strains was reported and proposed to be associated with the HMV phenotype (17). Moreover, binding of KP-M1 to Siglec-9 on the surface of neutrophils was found to dampen neutrophil activation and phagocytosis (17). Siglec-9 is a member of the immunomodulatory sialic acid-binding immunoglobulin-like lectin (Siglec) family (20, 21). These lectins are primarily found on the surface of immune cells and most of them are involved in inhibition of immune cell activation, which is exploited by some bacteria to evade immune control (22, 23). A few activating Siglecs (e.g. Siglecs 14 and 16) pairing inhibitory Siglecs (Siglec-5 and Siglec-11, respectively) have possibly evolved to counteract bacteria exploiting inhibitory Siglecs (23).

Using a similar molecular mimicry strategy, several bacteria display host-like galactose (Gal)-containing motifs to avoid recognition by innate immune lectins targeting non-self carbohydrate patterns (19). Different members of the galectin family selectively recognize bacterial strains expressing such host-like structures. In particular, several galectins have been found to bind *K. pneumoniae* strains and bactericidal activity towards strains displaying blood group-like antigens has been reported (24–27), although none of those strains exhibited the HMV phenotype. Of note, the O-antigen (or O-chain) was identified as the primary ligand for Gal-specific lectins, including galectins, on the surface of the *K. pneumoniae* strain 52145 (18, 25). The O-chain is the outermost part of the lipopolysaccharide, which is a main carbohydrate structure displayed on Gram-negative bacteria. To date, 12 *K. pneumoniae* O serotypes (including subgroups), built with different repeating saccharide units, have been described (28).

Fueled by all these precedents, in this work we have examined the sialic acid content of a collection of HMV and non-HMV *K. pneumoniae* clinical isolates and their recognition by several Siglecs. Analysis of sugar epitopes displayed on the bacterial surface, by exploring the binding of model lectins with diverse carbohydrate-binding specificities, unequivocally revealed the presence of Gal-containing motifs in many isolates, prompting the study of their recognition by different galectins. Finally, potential galectin ligands in the outer membrane were explored.

## 2 Results

### 2.1 Sialic acid content of *K. pneumoniae* isolates and recognition by Siglecs

Forty-one HMV and seven non-HMV *K*. *pneumoniae* isolates were examined. The isolates were classified into four groups: HMV *magA*+*rmpA*+ (group 1), HMV *magA*-*rmpA*+ (group 2), HMV *magA*-*rmpA*- (group 3), and non-HMV *magA*-*rmpA*- (group 4) (**Table 1**). The total sialic acid content of the isolates was determined by quantitation of sialic acid released from bacterial cells by hydrolysis. For most isolates, no major differences were observed, with values ranging from 7 × 10^-3^ to 14 × 10^-3^ μmol/10^9^ bacterial cell (median value 9 × 10^-3^ μmol/10^9^ bacterial cell) (**Supplementary Table 1**). Only for isolate 39, a visibly higher content of 24 × 10^-3^ μmol/10^9^ bacterial cell was noticed.

**Table 1:** *K. pneumoniae* clinical isolates.

Group	Isolate ID	Capsular type	O-type	MLST	Wzi Allele	Origin	Bacteremia Acquisition
1	1	K1	O1	23	1	Pneumonia	Nosocomial
	2	K1	ND	23	1	Liver abscess	Health care associated
	4	K1	O1	23	1	No focus	Health care associated
	5	K1	O1/O2	23	1	No focus	Health care associated
	8	K1	O1	23	1	Non urinary catheter	Health care associated
	9	K1	O1	23	1	Non urinary catheter	Community acquired
	11	K1	ND	23	1	Liver abscess	Health care associated
	12	K1	O1	23	1	Liver abscess	Health care associated
	13	K1	O1	23	1	Urinary catheter	Nosocomial
	15	K1	O1	23	1	No focus	Community acquired
	16	K1	O1	23	1	Liver abscess	Health care associated
	18	K1	ND	23	1	Unknown	Unknown
	19	K1	O1	23	1	Non urinary catheter	Health care associated
	20	K1	O1	23	1	Catheter	Nosocomial
	21	K1	O1	23	1	Spontaneous bacterial peritonitis	Health care associated
	23	K1	O1	23	1	Catheter	Nosocomial
	103	K1	ND	81	1	Respiratory	Nosocomial
2	3	K2	O1	25	72	Urinary catheter	Nosocomial
	6	noK1/K2	O1	1013	122	Urinary catheter	Nosocomial
	7	K2	O1	380	2	Pneumonia	Community acquired
	10	K2	O1/O2	380	2	Liver abscess	Health care associated
	17	K2	O1	86	2	Pneumonia	Community acquired
	22	K2	O1	380	2	Pneumonia	Health care associated
	24	K2	O1	86	2	Urinary catheter	Nosocomial
	25	K2	O1	86	2	Abdominal	Community acquired
	26	K2	O1	65	72	Abdominal	Community acquired
	27	noK1/K2	O1	1013	122	Liver abscess	Community acquired
	28	noK1/K2	O1	1013	122	Biliary	Health care associated
	29	K2	O1	493	2	No urinary catheter	Community acquired
3	14	K30	O1	416	138	Liver abscess	Health care associated
	30	noK1/K2	O1/O2	1035	116	Biliary	Health care associated
	31	noK1/K2	ND	895	173	Liver abscess	Health care associated
	32	noK1/K2	O4	895	173	Biliary	Community acquired
	34	K35	O1	460	162	Biliary	Community acquired
	35	noK1/K2	O3	tlv719	186	Biliary	Community acquired
	37	noK1/K2	ND	622	193	Biliary	Community acquired
	43	K24	O2	45	101	No urinary catheter	Health care associated
	44	K15K17K50K51K52	O4	37	50	Urinary catheter	Community acquired
	46	K26	O2	321	113	Catheter	Nosocomial
	47	K26	O3	17	113	Biliary	Community acquired
	102	noK1/K2	O4	152	110	Urinary catheter	Nosocomial
4	33	K22.37	O1	35	37	Biliary	Nosocomial
	36	K26	O2	17	113	Biliary	Community acquired
	38	K22.37	O1	35	37	Liver abscess	Community acquired
	39	K54	O3	214	115	Liver abscess	Nosocomial
	40	K31	O3	104	102	Pneumonia	Community acquired
	41	ND	O1	711	ND	Pneumonia	Nosocomial
	42	ND	O1	465	ND	Biliary	Health care associated

Based on the absence or presence of the HMV phenotype and magA and rmpA genes, the isolates are classified into four groups: 1, HMV magA+rmpA+; 2, HMV magA-rmpA+; 3, HMV magA-rmpA-; and 4, non-HMV magA-rmpA-. MLST, multilocus sequence type; ND, not determined.

**Figure 1:**
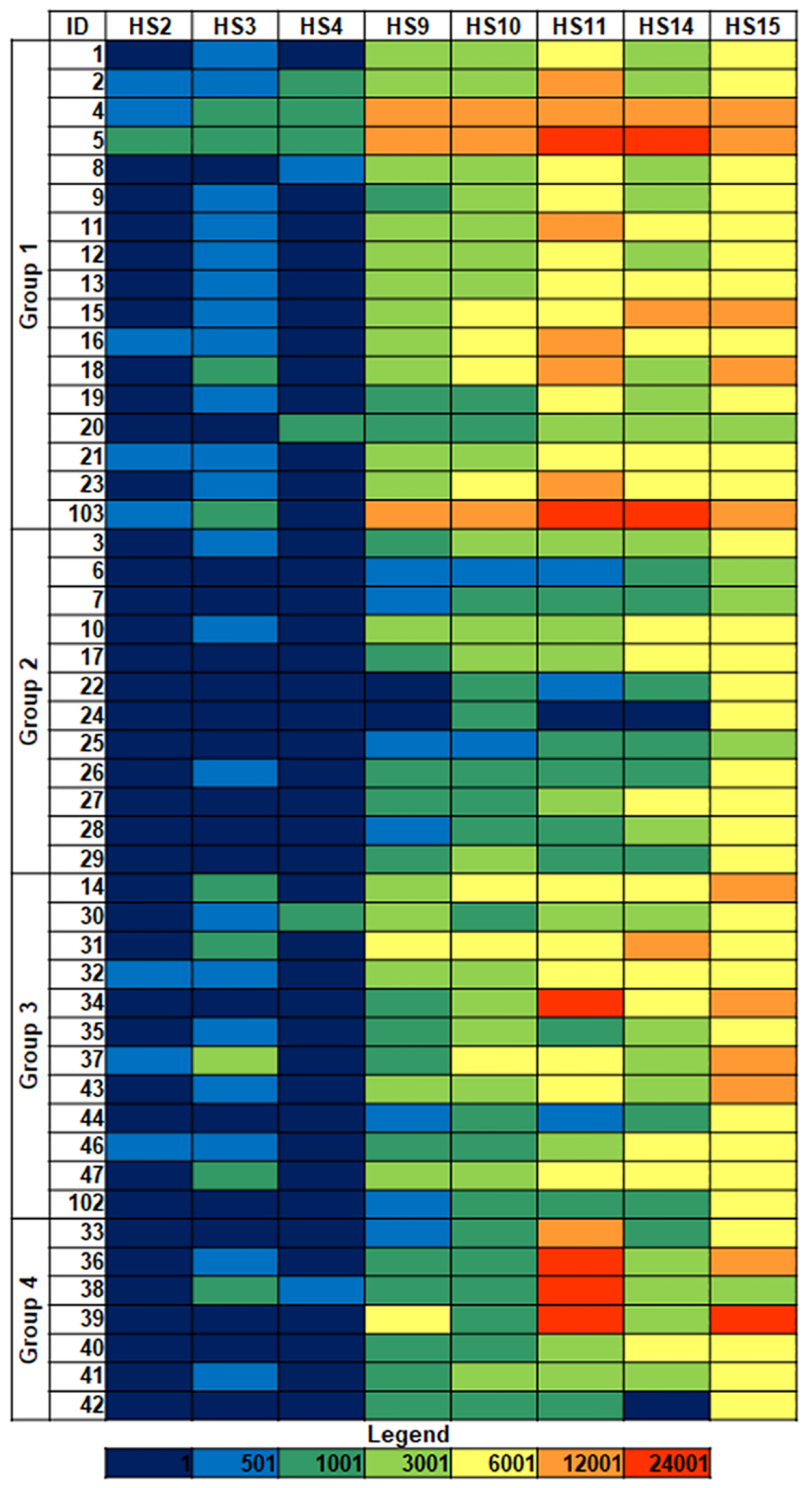
Siglec binding to *K. pneumoniae* isolates. Group 1: HMV *magA*+*rmpA*+; Group 2: HMV *magA*-*rmpA*+; Group 3: HMV *magA*-*rmpA*-; Group 4: non-HMV *magA*-*rmpA*-. Binding intensities to bacterial cells printed using suspensions with OD_600_ = 1 are shown. Fluorescence is represented using the following color code: dark blue, <500 relative fluorescence units (rfu); blue 501–1,000; dark green, 1,001–3,000; green, 3,001–6,000; yellow, 6,001–12,000; orange, 12,001–42,000; red, >24,001. HS, human Siglec.

Recognition of the isolates by different Siglecs, including evolutionarily conserved Siglecs (namely, Siglecs 2, 4, and 15), and CD-33-related Siglecs (3, 9, 10, 11, and 14) (23), was examined in binding assays to microarray-printed bacteria (**Figure 1**; **Supplementary Figure 1**). Based on fluorescence intensity, binding signals were catalogued into three main levels: weak (colored in blue/dark green in the heatmaps), moderate (colored in green/yellow), and intense (colored in orange/red). Binding signals detected for Siglecs 2, 3, and 4 were weak or even negligible (colored in dark blue). On the other hand, isolate- and Siglec-dependent binding was observed for Siglecs 9, 10, 11, 14, and 15 (**Figure 1**). Interestingly, only weak to moderate binding of Siglecs 9 to 15 to isolates 20 (group 1), 6, 7, and 25 (group 2), all of them exhibiting the HMV phenotype, was detected. Furthermore, a markedly selective recognition of several isolates by particular Siglecs was observed (see e.g. isolate 34). From the Siglecs’ perspective, no clear-cut differences in the binding patterns to isolates belonging to the different groups were evident. In general, more intense binding signals were detected for Siglecs 11, 14, and 15, with Siglec-15 frequently showing the strongest binding intensities. Of note, the ligand-contact surface of Siglec-15 is larger than for other Siglecs (29), which could contribute to a stronger binding.

### 2.2 Exploration of sugar epitopes on the surface of *K. pneumoniae* isolates

**Figure 2:**
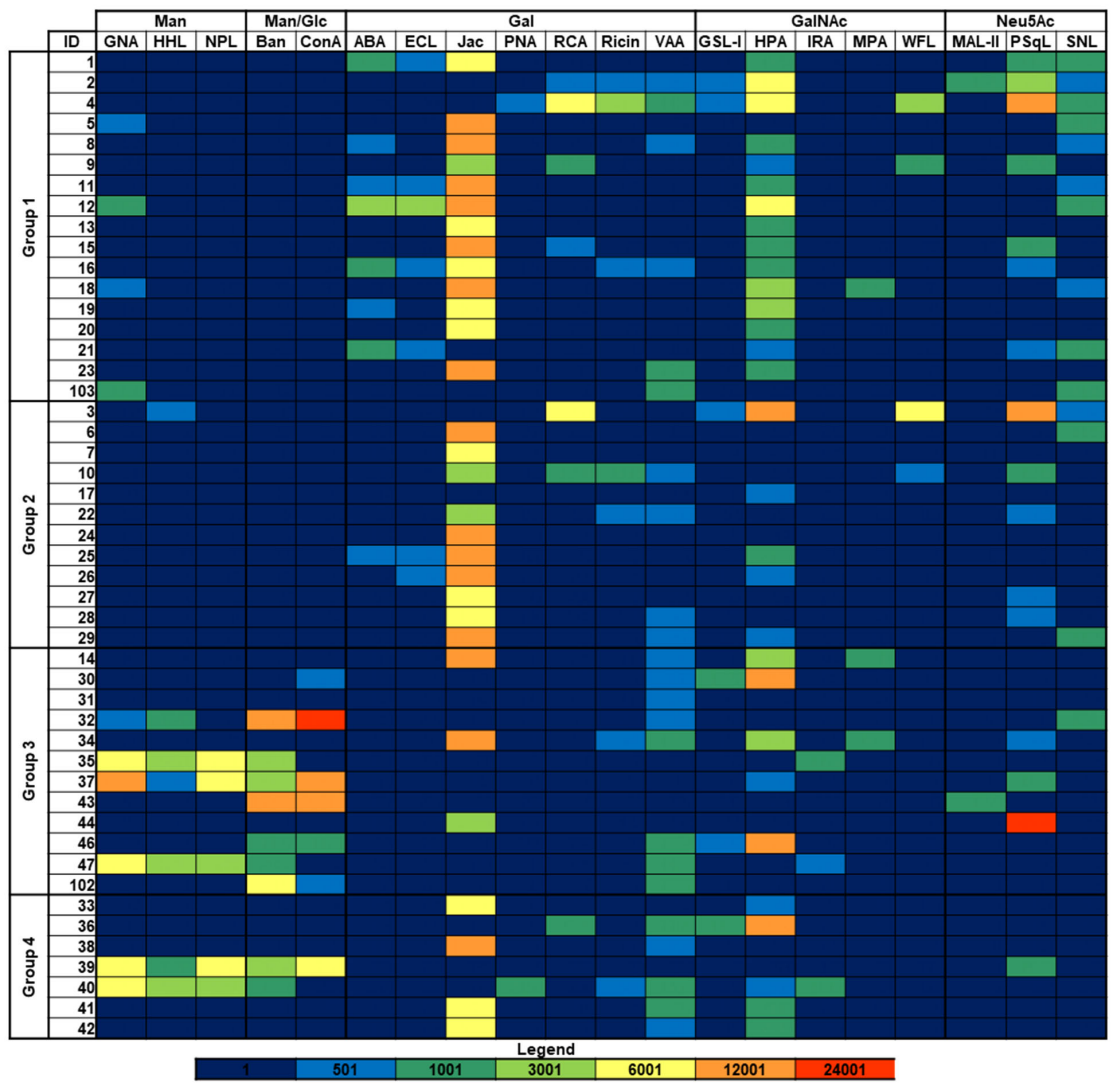
Binding of model lectins to *K. pneumoniae* isolates. Group 1: HMV *magA*+*rmpA*+; Group 2: HMV *magA*-*rmpA*+; Group 3: HMV *magA*-*rmpA*-; Group 4: non-HMV *magA*-*rmpA*-. Binding intensities to bacterial cells printed using suspensions with OD_600_ = 1 are shown. Abbreviations used for lectin names are defined in **Supplementary Table 2** except that Ban and Jac state for BanLec and Jacalin, respectively. Fluorescence is represented using the same color code as in **Figure 1**.

**Figure 3:**
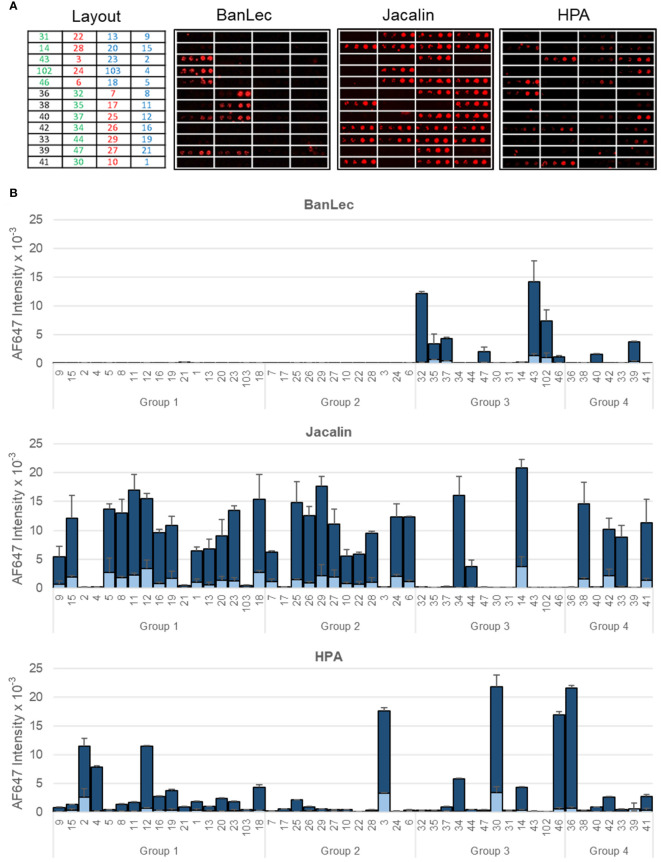
Binding of representative model lectins to *K pneumoniae* isolates. Group 1: HMV *magA*+*rmpA*+; Group 2: HMV *magA*-*rmpA*+; Group 3: HMV *magA*-*rmpA*-; Group 4: non-HMV *magA*-*rmpA*-. **(A)** Microarray images obtained for the binding to isolates printed as duplicates at OD_600_ of 0.3 and 1. The layout indicates the position of the isolates in the array. **(B)** Quantitated fluorescence intensities. Dark blue, binding to bacteria printed at OD_600_ = 1; light blue, binding to bacteria printed at OD_600_ = 0.3. Data shown correspond to mean values of duplicate signal intensities and error bars indicate the standard deviation to the mean.

Besides sialic acid, the presence of other sugar epitopes on the bacterial surfaces was explored by examining the binding to microarray-printed bacteria of a panel of 34 model lectins with diverse carbohydrate-binding specificities (**Supplementary Table 2**). Binding signals were only detected for 20 of the lectins tested (**Figure 2**; **Supplementary Figure 2**). Microarray images and quantitated dose-dependent binding intensities obtained for three representative lectins are shown in **Figure 3**. As could be expected, recognition by the Neu5Ac (sialic acid)-specific lectins PSqL and/or SNL (see **Supplementary Table 2** for definition of abbreviations) was observed for diverse isolates. On the other hand, intense binding signals for mannose (Man)- or Man/glucose (Glc)-specific lectins, as BanLec (**Figure 3**), were only observed for several isolates belonging to groups 3 and 4 (**Figure 2**). In contrast, the Gal-specific lectin Jacalin bound strongly to many isolates from the four groups, with the remarkable exception of those isolates showing intense binding signals of Man/Glc-specific lectins (**Figures 2**, **3**). A third conspicuous lectin was the *N*-acetyl-galactosamine (GalNAc)-specific lectin HPA, which also gave binding signals for many isolates from the four groups, with a binding pattern rather comparable to that of Jacalin. Of note, besides glycans with terminal GalNAc moieties, HPA is able to recognize the Thomsen-Friedenreich antigen Galβ1,3GalNAc, which is a well-known Jacalin ligand (30, 31) and also a binding partner for different galectins (32). Thus, the results of the glycotyping analysis pointed to the presence of potential galectin ligands on many isolates, prompting the study of their recognition by different members of this lectin family.

### 2.3 Recognition of *K. pneumoniae* isolates by galectins

**Figure 4:**
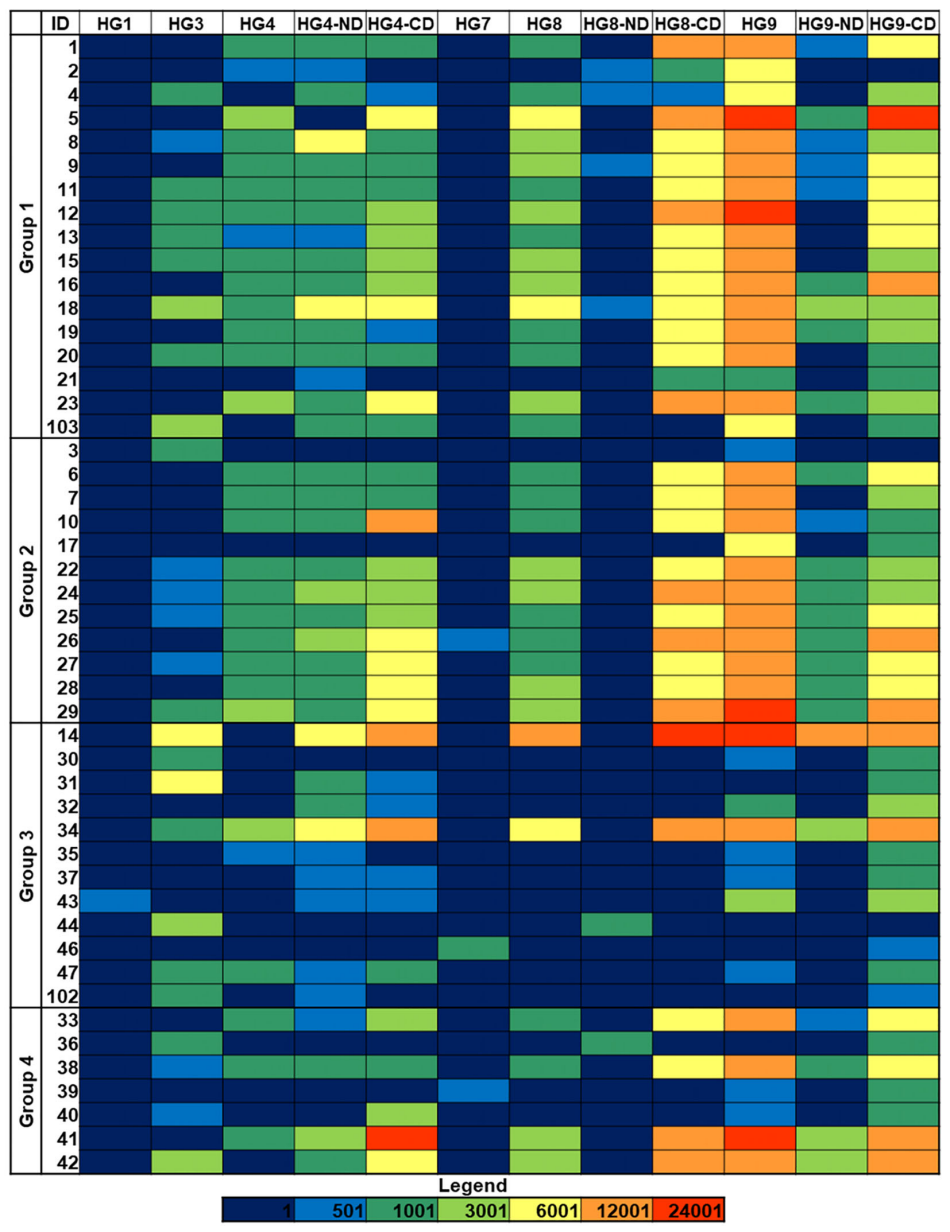
Galectin binding to *K. pneumoniae* isolates. Group 1: HMV *magA*+*rmpA*+; Group 2: HMV *magA*-*rmpA*+; Group 3: HMV *magA*-*rmpA*-; Group 4: non-HMV *magA*-*rmpA*-. Binding intensities to bacterial cells printed using suspensions with OD_600_ = 1 are shown. Fluorescence is represented using the same color code as in **Figure 1**. HG, human galectin.

The binding to the isolates of six galectins belonging to the three structural subgroups of this family, namely galectins 1 and 7 (proto type, composed of two identical carbohydrate-recognition domains (CRDs) that form non-covalent homodimers), galectin-3 (chimera type, composed of one CRD linked to a non-lectin N-terminal region), and galectins 4, 8, and 9 (tandem-repeat type, containing two different CRDs covalently connected by a linker peptide) was examined. The behavior of the separate N- and C-terminal CRDs of galectins 4, 8, and 9 was also evaluated. With very few exceptions, no binding was detected for galectins 1 and 7 (**Figure 4**; **Supplementary Figure 3**), suggesting the absence of terminal Lac/LacNAc epitopes, which are the main ligands for these two galectins (33). Weak to moderate isolate- and galectin-selective binding was typically observed for galectins 3, 4, and 8, while galectin-9 displayed the most intense binding signals for many isolates. The comparison of the relative binding behavior of the separate N- and C-terminal CRDs of galectins 4, 8, and 9 evidenced a leading involvement of the C-CRD over the N-domain for the three galectins. In line with the behavior exhibited by Jacalin (see the previous section), no or very weak binding to those isolates recognized by Man/Glc-specific lectins was detected for any of the full-length proteins or separate CRDs. Further differences in the binding patterns of a given galectin to isolates belonging to the same group were visible, even among isolates with the same capsular and sequence type, as those of group 1 (**Figure 4**; **Table 1**). For galectin-9 in particular, a weaker binding was detected for isolates 2, 4, and 21 compared to the other isolates with serotype K1 and sequence type 23. Of note, these three isolates were not recognized by Jacalin (**Figure 2**), clearly indicating a significant variation in the structure or availability of Gal-containing structures.

### 2.4 Western blot analysis of potential ligands for galectin-9

For this analysis, two K1/ST23 isolates exhibiting different Gal-specific lectin binding patterns were selected: isolate 2, which was moderately bound by galectin-9 and not recognized by Jacalin, while gave significant binding signals with HPA (**Figure 2**; **Supplementary Figure 2**), and isolate 16, which was more intensely bound by galectin-9 and also recognized by Jacalin, while only gave weak binding signals for HPA, further pointing to differences in Gal-containing structures.

**Figure 5:**
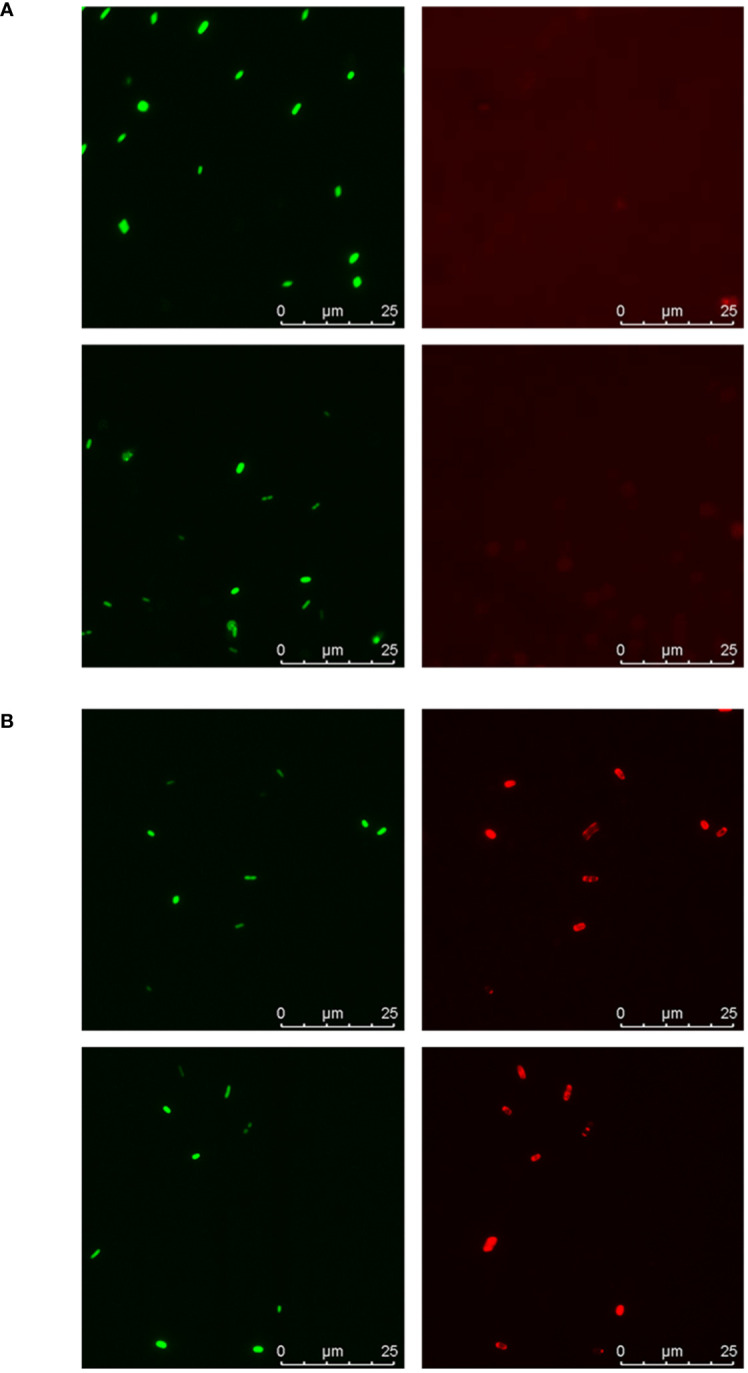
Fluorescence microscopy analysis of galectin-9 binding to isolates 2 and 16. Suspensions of SYTO-13-labelled bacterial cells at OD_600_ = 0.5 were incubated with 20 µg/mL of galectin-9 and bound galectin was detected following the same protocol used in the microarray-binding assays, with incubation with AF647-streptavidin as final step. Representative images obtained for isolate 2 **(A)** and isolate 16 **(B)**. Green: SYTO-13 signals. Red: AF647 signals. For each image, scale bars are shown.

First, fluorescence microscopy was used to visualize the binding of galectin-9 to the bacterial cells in suspension (**Figure 5**). SYTO-13-stained bacteria were seen with the blue filter (depicted in green) while bound galectin was detected with the red filter. No binding was observed for isolate 2 (**Figure 5A**), while for isolate 16 red signals co-localizing with bacterial cells were clearly visible (**Figure 5B**), thus confirming the binding trends observed in the microarray binding assays.

**Figure 6:**
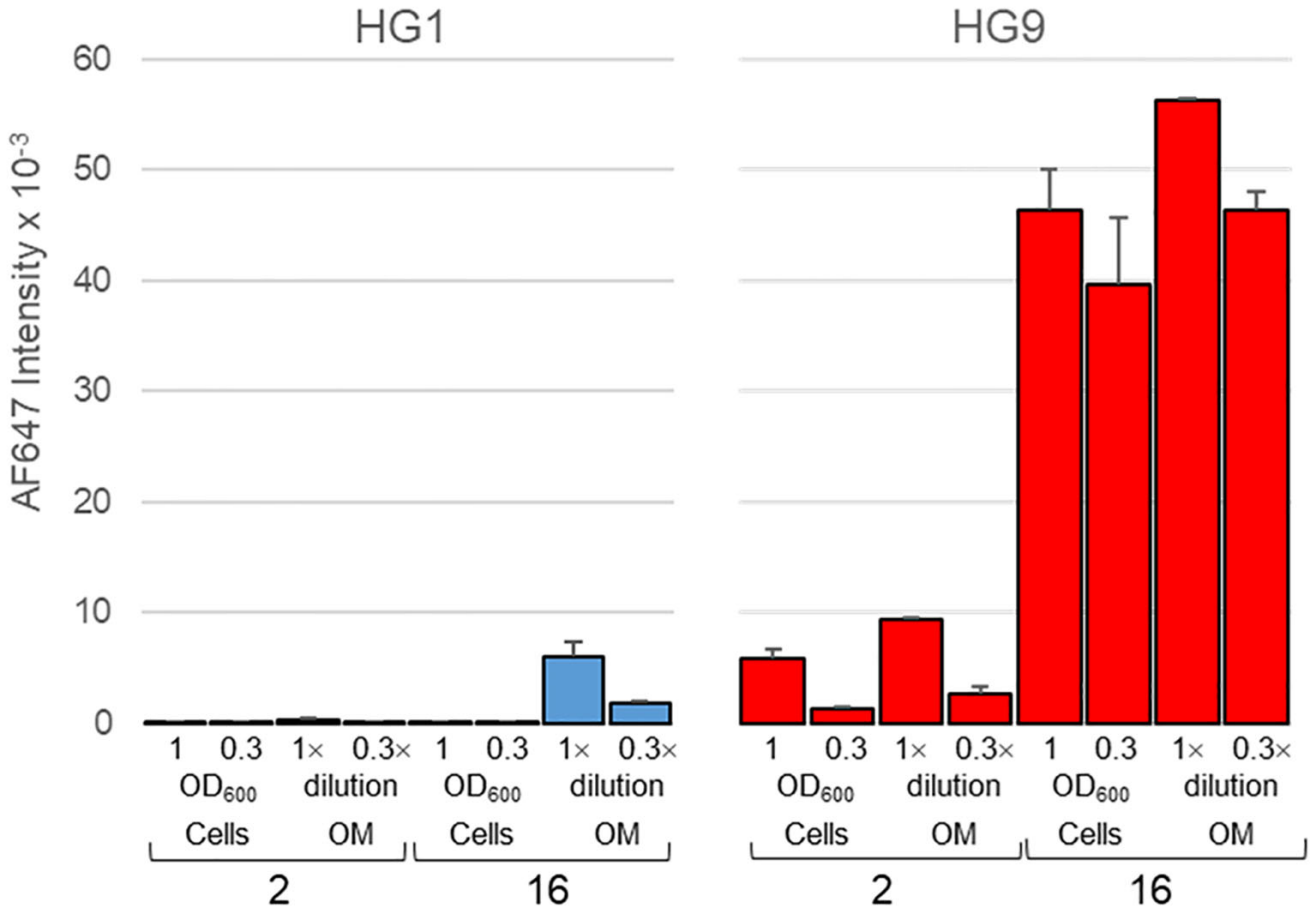
Microarray analysis of the binding of galectins 1 and 9 to outer membranes of isolates 2 and 16. Outer membranes (OM), along with their parental bacterial cells, were manually printed as duplicates at two different concentrations, namely, OD_600_ = 1/0.3 for cells and 1×/0.3× dilution of the stock preparation for OMs. Data shown correspond to mean values of duplicate signal intensities and error bars indicate the standard deviation to the mean.

The outer membrane of both isolates was next isolated and manually printed into microarray slides, along with their parental bacterial cells. Binding assays with galectin-9 proved recognition of outer membranes with intensities and binding patterns comparable to those observed for entire bacterial cells, that is, with an obvious preference for isolate 16 over isolate 2 (**Figure 6**). In contrast, only weak binding of galectin-1 to the outer membrane of isolate 16 was detected, even though binding signal intensities in manual arrays are much higher than in robotic arrays due to the greater amount of printed probe (34). This result also served as indicator of the absence of spurious terminal Gal-containing structures in the membrane preparations.

**Figure 7:**
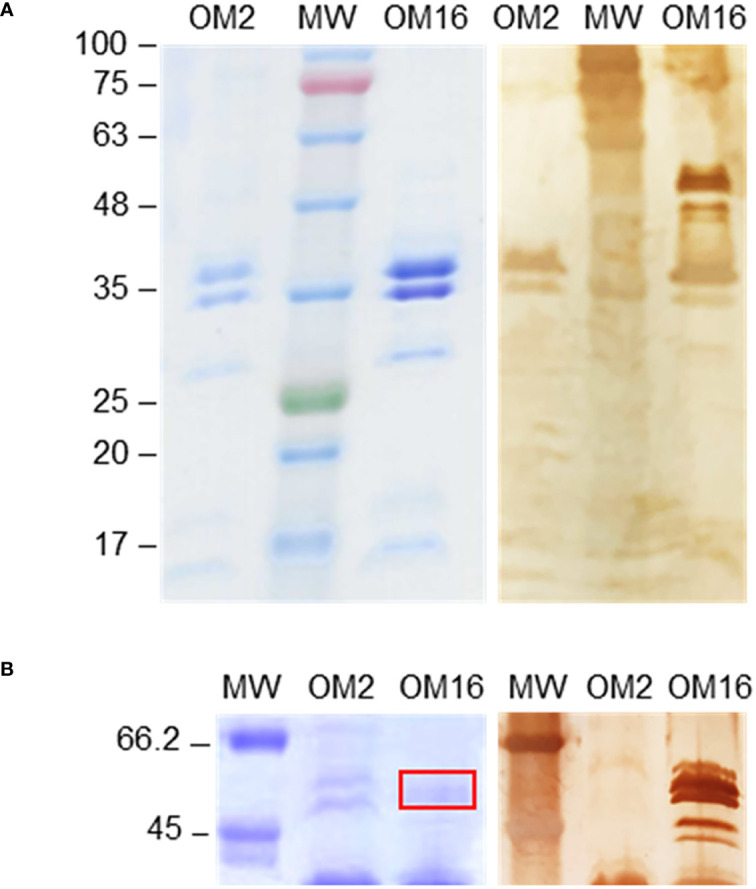
Western blot analysis of the binding of galectin-9 to outer membranes of isolates 2 and 16. Outer membrane (OM) samples were run in 10% polyacrylamide gels under reducing conditions, along with molecular weight markers (MW), and protein bands were either stained with Coomassie Brilliant Blue (left panels) or transferred to a nitrocellulose membrane. The binding of biotinylated galectin-9 was examined using horseradish peroxidase-conjugated streptavidin and 3,3’-diaminobenzidine for final chromogenic detection (right panels), as detailed in Materials and methods. **(A)** Coomassie Blue-stained gel and Western blot results obtained using 5 µL of OM stock preparation. **(B)** Gel and blot region of galectin-9-positive bands detected for isolate 16 but not for isolate 2 using 20 µL of OM stock preparation. Bands excised from the gel for fingerprint analysis are signaled by a red rectangle. The electrophoretic mobility and molecular weight (in kDa) of markers (pre-stained in panel **A**) is indicated on the left.

Possible glycoprotein ligands for galectin-9 in the outer membrane were then examined by Western blot and fingerprint analyses. Similar major protein bands were observed for both isolates in Coomassie blue-stained gels (**Figure 7A**). According to electrophoretic mobility and fingerprinting, these bands were compatible with porins OmpC and OmpA. However, in the Western blot analysis, strong galectin-9-binding signals were primarily observed for a group of bands with electrophoretic mobilities between 63–48 kDa, according to MW markers, and hardly visible by Coomassie blue staining. These galectin-9-positive bands were detected for isolate 16 but not for isolate 2, even when examining heavily overloaded blots (**Figure 7B**), and were scarcely visible when the outer membrane preparation was treated with proteinase K before electrophoresis (**Supplementary Figure 4**), pointing to protein digestion. Of note, these bands were not recognized by Siglec-10 in a similar Western blot analysis (not shown).

Fingerprint analysis by nanosystem liquid chromatography-tandem mass spectrometry (nLC MS/MS) was used to identify proteins migrating at this position. A minimum threshold of 2 peptide hits and 10% sequence coverage was set for considering positive identification, and the results were further filtered based on molecular weight and known protein localization, finally yielding a list of six outer membrane proteins (**Table 2**). The highest score was obtained for TolC, which is a minor but functionally important membrane protein involved in secretion and efflux systems (35). To the best of our knowledge, glycosylation of TolC or of the other identified proteins has not been reported to date. Still, using GlycoPP, a webserver for predicting potential *N*- and *O*-glycosites in prokaryotic protein sequences (36), three *O*-glycosylation sites in the extracellular loop of the protein (37), namely Ser279, Thr281, and Ser297, were predicted, and the same was found for the sucrose porin (ScrY) and the capsule assembly Wzi family protein, which also contain potential *O*-glycosites within the extracellular loops. Thus, it is plausible that these sites could display Gal-containing glycans recognized by galectin-9. Nevertheless, other glycoproteins not spotted in the fingerprint analysis could contribute to the galectin-9 positive signals.

**Table 2:** Fingerprint analysis of potential glycoprotein ligands for galectin-9.

Accession	Description	Score	Coverage	# Proteins	# Unique Peptides	# Peptides	# PSMs	# AAs	MW [kDa]	Calc. pI
YP_005228876.1	Outer membrane channel protein [*K. pneumoniae* subsp. *pneumoniae* HS11286]	198,38	58,49	1	24	24	113	489	53,0	6,19
YP_005225626.1	Sucrose porin [*K. pneumoniae* subsp. *pneumoniae* HS11286]	43,24	25,35	2	12	12	29	505	55,6	5,92
YP_005227870.1	Capsule assembly Wzi family protein	38,35	17,82	1	9	9	28	477	52,8	5,68
YP_005228668.1	Putative outer membrane efflux protein MdtP [*K. pneumoniae* subsp. *pneumoniae* HS11286]	21,97	21,58	1	9	9	14	482	52,9	8,10
YP_005226686.1	Putative outer membrane efflux protein [*K. pneumoniae* subsp. *pneumoniae* HS11286]	20,61	19,17	1	9	9	13	459	50,0	6,10
YP_005224560.1	Maltoporin [*K. pneumoniae* subsp. *pneumoniae* HS11286]	3,45	16,78	1	6	6	7	429	47,8	4,94

Accession: protein accession number; Description: protein description; Score: protein score (sum of scores of individual peptides); Coverage: % coverage of the protein sequence; # Proteins: number of identified proteins; # Unique peptides: number of peptides unique to the protein; # Peptides: number of peptides identified in the protein; # PSMs (peptide spectrum matches): number of times peptides are detected; # AAs, MW, Calc. pI: calculated parameters for the protein based on amino acid sequence.

## 3 Discussion

The presence of diverse lectin-accessible carbohydrate epitopes on the surface of *K. pneumoniae* clinical isolates has been evidenced by scrutinizing their interactions with a panel of model lectins with defined carbohydrate-binding specificities, and their recognition by several members of the Siglec and galectin families. A larger sialic acid content, previously proposed to be associated with the HMV phenotype, was not observed for HMV isolates compared to non-HMV isolates. Moreover, no obvious differences in Siglec binding patterns between HMV and non-HMV isolates were detected. On the other hand, HMV isolates exhibited clearly disparate binding signals for Gal- and Man/Glc-specific model lectins. In particular, the Gal-specific lectin Jacalin bound strongly to many isolates from the four groups, with the remarkable exception of those isolates showing intense binding signals for Man/Glc-specific lectins. A similar behavior was observed for the GalNAc-specific lectin HPA.

The lectin-binding patterns did not correlate with the capsular serotype. In fact, intense binding of Gal/GalNAc-specific lectins to many K1 and K2 isolates was observed (K serotypes for each isolate are specified in **Table 1**), although the polysaccharide capsule of these serotypes contains Glc and/or Man residues but not Gal (38). In contrast, the same lectins did not bind to K26 or K31 isolates, which do contain Gal in their capsule (38). Strikingly, binding signals of Man/Glc-specific lectins to K1 and K2 isolates and other isolates with polysaccharide capsules containing Man residues, as K24, K26, or K31, were weak or even negligible, while these lectins did bind to isolates with capsular serotypes not reported to contain Man moieties, as K32, K39, and K40 (38). Thus, these lectins must recognize other surface carbohydrate structures.

A possible binding candidate is the O-antigen (or O-chain). Of the 12 *K. pneumoniae* O serotypes described to date (28), serotypes O1, O2, O3, and O5 have been found to be the most prevalent, accounting on average for 48.5%, 18%, 15%, and 8.5% of two large multi-country isolate collections studied (39, 40). O1 and O2 antigens contain a repeating D-galactan unit, while O3 and O5 are composed of D-Man units. The O-chain of the O1:K2 *K. pneumoniae* strain 52145 was identified as the primary ligand on the bacterial surface for different galectins (25). Moreover, the K1 and K2 capsule types are frequently associated with the O1 antigen. Altogether, it seems plausible that O1/O2 chains on *K. pneumoniae* isolates can serve as ligands for Gal-specific lectins, while O3/O5 chains can be recognized by Man-specific lectins. Indeed, O-typing identified the O1/O2 chain in all the isolates of groups 1 and 2 examined (**Table 1**). Moreover, the O3 type was identified in four isolates of groups 3 and 4, namely isolates 35, 39, 40, and 47 (**Table 1**). The repeating unit of the O3 antigen is Manα(1,2)Manα(1,2)Manα(1,3)Manα(1,3)Man (41), which can serve as ligand for Man-specific lectins. Fully in agreement, the four isolates with typed O3 chains gave intense binding signals for Man-specific lectins.

Similarly, our results strongly suggest that the O1/O2-chains could also serve as galectin ligands, while this is not the case for Siglecs. Indeed, these sialic-acid-binding lectins did not show clear-cut differences in their binding patterns to isolates belonging to the four *K. pneumoniae* groups or displaying different typed O-antigens. Moreover, galectin- and Siglec-binding patterns did not correlate. Interestingly, some isolates showed almost exclusive binding of Siglecs, others displayed predominant binding of galectins, while a third set of isolates exhibited moderate to intense binding signals for several Siglecs and galectins (**Supplementary Figure 5**). It is worth mentioning that, besides sialic acid-containing structures, several Siglecs have been reported to bind other sugars and various non-sialylated ligands through protein-protein or protein-lipid interactions (42). Relevant examples of such ligands in pathogens include the β protein from group B *Streptococcus*, which is not glycosylated (43), lipids of the dermatophytic fungus *Trichophyton* (44), and the high molecular weight hyaluronic acid expressed by group A *Streptococcus*, which consists of repeating GlcNAcβ(1,4)GlcAβ(1,3) units (GlcA standing for glucuronic acid) (45). Fittingly, GlcA has been found in the repeating unit of most *K. pneumoniae* K types (46–52), and the amount of GlcA has been reported to be inversely proportional to the rate of phagocytosis by neutrophils (53). An analogous phenomenon has been described for the sialic acid-rich *K. pneumoniae* isolate KP-M1 upon binding to Siglec-9 on the neutrophils’ surface (17). Engaging inhibitory Siglecs via recognition of GlcA could be similarly exploited to prevent immune cell activation. Therefore, although interactions with other surface structures could be possible, it is tempting to speculate that capsular GlcA might be recognized by several Siglecs, thus explaining the wide-ranging recognition of most of the isolates herein examined.

Besides the O-chain, the presence of other ligands for galectins has been put forward in a previous study, in which significant galectin binding to a double mutant of *K. pneumoniae* strain 52145 devoid of O-chain and capsule was observed (25). Although, to the best of our knowledge, *K. pneumoniae* glycoproteins have not been described to date, the Western blot analysis carried out herein to scrutinize the binding of galectin-9 to outer membrane proteins revealed several protein bands that were recognized by this galectin. Moreover, the analysis of potential glycosites in the proteins identified by fingerprint analysis predicted possible *O*-glycosylation sites. Indeed, different *O*-glycosylation mechanisms have been described in numerous and diverse bacterial species, including various important human pathogens (54, 55). In particular, *O*-oligosaccharyltransferases (*O*-OTases), which have been found in many Gram-negative bacteria, can transfer the building blocks used in capsule or O-antigen biosynthesis and often target multiple proteins (54–60). Altogether, considering that the binding of galectin-9 to protein bands in the Western blot analysis correlates with the binding to outer membranes and also to entire cells, it is likely that *K. pneumoniae* shares with other Gram-negative bacterial species the ability to glycosylate outer membrane proteins with O-antigen building blocks. Nonetheless, more data are definitely required to unambiguously establish the occurrence of *K. pneumoniae* glycoproteins.

Overall, the research described herein demonstrate that Siglecs and galectins apparently target different structures on *K. pneumoniae* surfaces, with likely distinct consequences in disease establishment and progression. Globally, this work illustrates the diversity of recognition events that innate immune lectins can establish with infectious bacteria and may open new research avenues in the fight against infections.

## 4 Materials and methods

### 4.1 *K. pneumoniae* clinical isolates

*K*. *pneumoniae* isolates were collected from blood samples in the Bellvitge University Hospital between 2007 and 2013 (13). The hypermucoviscous phenotype was identified using the string test and genes *magA* and *rmpA* were detected by PCR. The isolates were cataloged as HMV *magA*+*rmpA*+ (group 1, 17 isolates), HMV *magA*-*rmpA*+ (group 2, 12 isolates), HMV *magA*-*rmpA*- (group 3, 12 isolates), and non-HMV *magA*-*rmpA*- (group 4, 7 isolates) (**Table 1**). O-typing was also carried out by PCR (61).

Bacterial cells were grown in BHI, fixed with 4% formaldehyde, and labelled with SYTO-13 (Invitrogen) as previously described (25). The labelling efficiency was assessed by measuring the fluorescence intensity of bacteria suspensions at OD_600_ = 1 in 10 mM Tris/HCl, pH 7.8, 0.15 M NaCl (TBS), using a Horiba Jobin Yvon Fluoromax-4 spectrofluorometer. Total sialic acid content of the isolates was determined following incubation at 80 °C for 60 min with the Hydrolysis Reagent of the Sialic Acid Assay Kit (Sigma Aldrich). Released sialic acid was next oxidized to formylpyruvic acid, which finally reacted with thiobarbituric acid to form a colored product that was quantitated both colorimetrically (549 nm) and fluorimetrically (Λ_exc_ = 555/Λ_em_ = 585 nm), yielding equivalent results.

For isolation of outer membranes, cells were harvested by centrifugation of 90 mL of bacterial culture at OD_600_ = 0.8–0.9 and lysed by sonication. Unbroken cells were removed by centrifugation for 5 min at 5000 × *g* and the membrane fraction was next pelleted by centrifugation for 1 h at 100000 × *g* (62). To separate inner and outer membranes, the membrane fraction was resuspended in 1.5% (v/v) Triton X-100, incubated at 4 °C for 30 min, and centrifuged at 100000 × *g* for 30 min (63). The pellet, containing the outer membrane, was washed with 5 mM sodium phosphate, pH 7.2, 0.2 M NaCl (PBS) and finally resuspended in 500 μL of the same buffer (stock preparation).

### 4.2 Siglecs and galectins

Fc-tagged human Siglecs 2, 3, and 4 were obtained from Sino Biological (catalog numbers 11958-H02H, 12238-H05H, and 13186-H02H), while Siglecs 9, 10, 11, 14, and 15 were from R&D Systems (catalog numbers 1139-SL, 2130-SL, 3258-SL, 4905-SL, and 9277-SL). Human galectins 1, 3, 7, 8, and 9, and the separate N- and C-CRDs of galectins 4, 8, and 9, were produced in BL21 *E. coli* cells via induction with isopropyl β-D-1-thio-galactopyranoside, while *E. coli* Rosetta (DE3) cells were used for galectin-4, as previously described (64–67). Briefly, following cell lysis, galectins 1, 3, 7, 8, 9, and the N-domain of galectin-8 were purified by affinity chromatography on lactose-agarose (Sigma-Aldrich), using 0.1 M lactose-containing buffer as eluant and, after extensive dialysis of the eluted fraction, the absence of lactose was confirmed by NMR spectroscopy. Galectin-4, its isolated N and C domains, and the C domain of galectin-8 were purified through Ni-NTA affinity chromatography followed by size exclusion chromatography in Superdex 75 columns. For galectin-9, pellets were resuspended in 10 mL of lysis buffer/g of pellet (lysis buffer being 10 mM Tris-HCl, pH 7.5, 0.5 M NaCl, 1 mM PMSF, 1 mM DTT). After sonication in ice, the crude extract was mixed with 1% Triton for 30 min at 4 °C and later clarified by ultracentrifugation. The soluble fraction was loaded onto 5 mL Glutathione-Sepharose resin previously equilibrated with TBS buffer. After centrifugation at 1.5 × *g* for 5 min, the resin loaded was washed with 50 mL of TBS containing 0.03% CHAPS, until the absorbance was zero. Then, the resin was incubated overnight with 25 µL thrombin/mL resin and 5 mL of PBS at 4 °C. The flow-through was finally loaded into a gel filtration Superdex 75 column and eluted with PBS containing 1 mM DTT and 0.1% NaN_3_. All galectin constructs were synthesized by GenScript, except those of galectin-9, which were obtained from the Riken BioResource Research Center (cat# RDB08416, RDB08423 and RDB08424). Galectins’ purity was checked by polyacrylamide gel electrophoresis (PAGE) (**Supplementary Figure 6**) and LC-MS.

Galectins were biotinylated using biotin-6-aminohexanoic acid-*N*-hydroxysuccinimide ester (Biovision Inc), following the manufacturer instructions, in the presence of 20 mM lactose to prevent modification of amino groups of the carbohydrate-binding site. After biotinylation, galectins were again exhaustively dialyzed to remove lactose.

### 4.3 Microarray binding assays

Bacterial cells and control glycoproteins were printed on 16-pad nitrocellulose-coated glass slides (Grace Biolabs ONCYTE NOVA) using a non-contact arrayer (Sprint, Arrayjet Ltd.), essentially as described (34, 68). Probes were printed as duplicates at two different concentrations (OD_600_ = 1 and 0.3). Outer membranes, along with their parental bacterial cells, were printed on 4-pad slides (FAST-slides, Whatman) using a manual glass-slide arraying system (V&P Scientific) (34). The Cy3 dye (GE Healthcare) was included in control glycoprotein solutions and membrane suspensions to enable post-array monitoring of the spots (69). The microarrays were scanned with a GenePix 4200-AL scanner (Axon, Molecular Devices) for SYTO-13 and Cy3 signals using excitation wavelengths of 488 nm and 532 nm, respectively, and also at 635 nm as control of absence of signal, and stored in a dry dark place until used. Fluorescence signals were quantified with the GenePix Pro 6.0 or 7.0 software (Molecular Devices).

For binding assays, microarrays were first blocked for 1 h with 0.25% (v/v) Tween-20 in PBS, then rinsed with PBS and overlaid for 1.5 h with a 20 µg/mL solution of the different lectins in PBS (or TBS for calcium-dependent lectins, see **Supplementary Table 2**), containing 0.1% (v/v) Tween-20 and also 4 mM β-mercaptoethanol in the case of galectins (overlay buffer). Fc-tagged Siglecs were pre-complexed with biotinylated goat anti-human IgG antibody (Vector) at a 1:2 Siglec:antibody ratio. After 4 washes with PBS/TBS, the slides were overlaid for 35 min with Alexa Fluor 647 (AF647)-labelled streptavidin (Invitrogen) at 1 µg/mL in overlay buffer. All the steps were carried out protected from light and at 20°C. Finally, the slides were first washed thoroughly with PBS and then with milliQ water, and scanned using excitation wavelengths of 635 nm (red channel) and 488 nm (blue channel). Fluorescence at 635 nm of blank spots of printing buffer alone was always below 500 rfu.

### 4.4 Fluorescence microscopy

Suspensions of SYTO-13-labelled bacterial cells at OD_600_ = 0.5 in PBS containing 2 mM DTT were incubated for 90 min in the absence or presence of biotinylated galectin-9 at 20 µg/mL final concentration. Following incubation, bacteria suspensions were centrifuged, washed once with PBS 1 mM DTT, and then incubated with 1 µg/mL AF647-streptavidin for 30 min. After washing again, the samples were examined by fluorescence microscopy. All the steps were carried out protected from light and at 20 °C. Images were captured on a Leica AF6000 LX system microscope (Mannheim, Germany) using a 100X 1.4 NA objective lens and incident light fluorescence with Hg 100 W mercury lamp and filter cubes L5 (blue), excitation filter BP 480/40 for SYTO-13 signals and Y5 (red), excitation filter BP 620/60 for AF647 signals. Detection ranges were set to eliminate crosstalk between fluorophores.

### 4.5 Western blot and fingerprint analysis

Outer membrane suspensions were mixed with an equal volume of RIPA lysis buffer (25 mM Tris-HCl, pH 7.6, 150 mM NaCl, 1% NP-40, 1% sodium deoxycholate, 0.1% SDS, Thermo Scientific), vigorously vortexed, incubated for 15 min at room temperature, and finally sonicated for 2 min. Where indicated, outer membranes of isolate 16 were next incubated in the absence or presence of 25 µg/mL of proteinase K (Roche, PCR grade), for 3 h at 37 °C, and the digestion was stopped by addition of PMSF (Sigma) at 5 mM final concentration. The samples were then subjected to SDS-PAGE (10% polyacrylamide) under reducing conditions and protein bands were next transferred to a nitrocellulose membrane using a semi-dry PowerBlot XL system (Invitrogen). After transfer, the membrane was blocked by incubation for 1 h with 0.25% Tween 20 in PBS, washed twice with PBS, and incubated for 2 h with 20 µg/mL of either biotinylated galectin-9 or Fc-tagged Siglec-10 pre-complexed with biotinylated goat anti-human IgG antibody (1:2 Siglec:antibody ratio) in PBS containing 0.1% Tween 20 and also 4 mM β-mercaptoethanol in the case of galectin-9. The membrane was then washed 4 times with PBS and incubated for 35 min with 1 μg/mL horseradish peroxidase-conjugated streptavidin (Invitrogen) in PBS containing 0.1% Tween 20. Finally, the membrane was washed 4 times with PBS and rinsed with milliQ water, and bound streptavidin was detected by incubating with 3,3’-diaminobenzidine and urea hydrogen peroxide (SigmaFast Tablets).

For fingerprint analysis, a second gel was run in parallel under identical conditions and stained with Coomassie Brilliant Blue. Bands positive for galectin-9 in the Western blot analysis were excised from the stained gel. After in-gel digestion of protein bands with porcine trypsin (Thermo Fisher Scientific), the generated peptides were examined by nLC–MS/MS. Full experimental details are given in the **Supplementary Materials and Methods** section. MS data were analyzed with Thermo Scientific Proteome Discoverer (version 1.4.1.14) using standardized workflows. Mass spectra *.raw files were searched against database NCBI, taxonomy *Klebsiella pneumoniae*, using the Sequest search engine. Precursor and fragment mass tolerance were set to 10 ppm and 0.02 Da, respectively, allowing 2 missed cleavages, carbamidomethylation of cysteines as a fixed modification, and methionine oxidation as a variable modification. Identified peptides were filtered using Percolator algorithm (70) with a q-value threshold of 0.01.

## Data availability statement

The data presented in this study are deposited in the FigShare repository, accession number 10.6084/m9.figshare.26206421.

## Author contributions

MC-R: Conceptualization, Formal analysis, Investigation, Validation, Visualization, Writing – review & editing. SM: Resources, Writing – review & editing. NH-O: Investigation, Writing – review & editing. MC: Resources, Writing – review & editing. JE-O: Resources, Writing – review & editing. AA: Resources, Writing – review & editing. JJ-B: Funding acquisition, Resources, Writing – review & editing. CA: Funding acquisition, Writing – review & editing. DS: Conceptualization, Formal analysis, Funding acquisition, Supervision, Writing – original draft, Writing – review & editing.
