# Supplementary material for: Insights into the recognition of hypermucoviscous Klebsiella pneumoniae clinical isolates by innate immune lectins of the Siglec and galectin families
Source: Front Immunol. 2024 Aug 1;15:1436039. doi: 10.3389/fimmu.2024.1436039 (PMC11324429; doi:10.3389/fimmu.2024.1436039)
Supplement: Supplementary file 1 [file DataSheet_1.pdf]

Supplementary Material

1 Supplementary Materials and Methods

Fingerprint analysis: For in-gel digestion of protein bands excised from Coomassie Blue-stained gels, these were cut into small pieces and destained with 50 mM ammonium bicarbonate and 50% acetonitrile. Samples were then reduced with 10 mM dithiothreitol in 50 mM ammonium bicarbonate, and alkylated with 55 mM iodoacetamide in 50 mM ammonium bicarbonate. Gel pieces were next digested with 12.5 ng/mL (final concentration) porcine trypsin in 50 mM ammonium bicarbonate, overnight at 37 °C. Peptides were extracted using 100% acetonitrile and 0.5% trifluoroacetic acid, purified using a Zip Tip (Millipore, Sigma-Aldrich), and dried. Finally, samples were reconstituted in 10 μL of 0.1% formic acid before analysis by nanosystem liquid chromatography-tandem mass spectrometry (nLC–MS/MS).

Peptide separations were carried out on an Easy-nLC 1000 nano system (Thermo Scientific). For the analysis, the sample was loaded into a precolumn Acclaim PepMap 100 (Thermo Scientific) and eluted in a RSLC PepMap C18, 50 cm long, 75 µm inner diameter and 2 µm particle size (Thermo Scientific). The mobile phase flow rate was 300 nL/min using 0.1% formic acid in water (solvent A) and 0.1% formic acid and 100% acetonitrile (solvent B). The gradient profile was set as follows: 2–40% solvent B for 30 min, 40–95% solvent B for 5 min, 95% solvent B for 5 min, 95%–0% solvent B for 20 min. Four microliters of each sample was injected. MS analysis was performed using a Q-Exactive mass spectrometer (Thermo Scientific). For ionization, 1900 V of liquid junction voltage and 300 °C capillary temperature was used. The full scan method employed a m/z 400–1500 mass selection, an Orbitrap resolution of 70,000 (at m/z 200), a target automatic gain control (AGC) value of 3e6, and maximum injection times of 100 ms. After the survey scan, the 15 most intense precursor ions were selected for MS/MS fragmentation.

Fragmentation was performed by Higher Energy Collisional Dissociation (HCD) with a normalized collision energy of 27 eV and MS/MS scans were acquired with a starting mass of m/z 200, AGC target was 2e5, resolution of 17500 (at m/z 200), intensity threshold of 8e4, isolation window of 2.0 m/z units and maximum IT was 100 ms. Charge state screening was enabled to reject unassigned, singly charged, and equal or more than seven protonated ions. A dynamic exclusion time of 20s was used to discriminate against previously selected ions.

2 Supplementary Figures and Tables

	ID	HS2	HS3	HS4	HS9	HS10	HS11	HS14	HS15
Group 1	1	368	531	230	3742	4144	10514	5072	9184
	2	557	544	1159	5061	5552	12106	3604	6849
	4	923	1824	1590	15197	13054	22694	19591	12761
	5	1600	1917	1049	16014	16359	29669	24523	23692
	8	121	368	716	3375	4415	8001	5480	8617
	9	221	501	20	2626	5917	7652	4449	8694
	11	184	622	222	4400	3285	13161	6899	9062
	12	198	585	0	3974	3478	10262	4909	8268
	13	293	589	488	3828	5187	8151	11443	9842
	15	80	824	304	5039	6650	11611	14108	12141
	16	802	913	310	5635	9145	13386	9610	11521
	18	214	1049	0	5734	7539	21826	5197	12902
	19	97	560	30	1927	1939	6506	4267	7919
	20	124	300	1264	1292	1656	5933	3165	4930
	21	838	727	0	3321	3674	7501	7880	10093
	23	0	843	444	5380	6007	16191	10233	11825
	103	930	2976	185	15364	18275	46654	24569	18452
Group 2	3	115	781	273	1942	4916	3763	5329	7841
	6	72	462	0	626	996	608	1324	5045
	7	70	201	0	870	1484	1254	1473	5413
	10	176	671	103	3171	3860	4176	7386	9951
	17	0	474	146	1599	3129	3270	7466	9487
	22	0	232	70	199	1499	980	2432	8201
	24	53	122	418	0	1873	0	0	8248
	25	55	275	0	938	751	1705	2343	5273
	26	88	510	0	1345	1785	1756	2470	9067
	27	0	401	173	1034	2623	3571	8416	8701
	28	0	349	124	561	2021	1486	5064	8114
	29	379	428	6	1845	3261	2375	2822	11042
Group 3	14	306	1818	0	4369	10502	9535	10720	15372
	30	136	787	1548	3335	2842	4551	4925	7012
	31	437	1553	223	8763	6698	9756	12826	10429
	32	820	808	210	5293	4584	7299	10527	10457
	34	139	474	344	2008	4107	24716	7127	21302
	35	303	716	0	1955	4866	2947	3190	9887
	37	558	3645	0	2570	6865	6966	5136	12859
	43	246	883	169	3995	3049	7810	5331	15594
	44	179	209	58	622	1229	996	1574	6231
	46	658	563	0	1951	2584	4019	7099	8549
	47	218	1246	214	4249	5299	7522	10092	7863
	102	214	180	432	953	2260	1787	2405	10762
Group 4	33	47	251	0	653	1807	23669	2271	10118
	36	151	622	88	2500	1812	45193	4287	17339
	38	171	1760	780	2003	1944	27446	4572	5604
	39	402	0	0	6283	2705	50294	5316	36642
	40	0	404	140	2806	2971	4119	7021	9550
	41	206	612	25	1912	4258	3329	5210	11927
	42	11	342	0	1631	2458	2192	493	7549

Supplementary Figure 1. Siglec binding to *K. pneumoniae* isolates. Fluorescence intensities to bacterial cells printed using suspensions with OD_{600nm} = 1 are shown. Data correspond to mean values of duplicate signal intensities. Median standard deviation was 10%. HS, human Siglec.

		Man			Man/Glc		Gal							GalNAc						Neu5Ac		
	ID	GNA	HHL	NPL	Ban	ConA	ABA	ECL	Jac	PNA	RCA	Ricin	VAA	GSL-I	HPA	IRA	MPA	WFL	MAL-II	PSqL	SNL
Group 1	1	449	62	46	55	70	1288	596	6489	0	112	304	498	164	1845	90	135	0	182	1105	2395
	2	273	325	60	79	231	184	196	124	127	572	888	952	616	11479	114	47	56	1346	3231	560
	4	350	362	30	55	140	390	200	144	793	7342	3072	1194	662	7840	143	55	5924	175	12355	2555
	5	562	124	57	61	134	232	241	13645	43	224	84	388	443	452	144	170	60	6	384	1000
	8	463	64	47	30	104	992	412	12956	20	131	340	761	207	1454	111	139	42	63	397	537
	9	100	74	19	27	41	137	154	5438	7	1536	125	355	122	861	53	58	1157	306	2347	75
	11	343	0	47	24	50	983	513	16927	20	51	165	171	130	1779	84	120	28	0	88	700
	12	1180	0	107	65	77	4390	3100	15466	28	0	0	184	190	11515	260	252	27	0	86	2013
	13	118	0	15	21	21	61	112	6774	26	37	59	0	97	1021	94	78	0	0	94	0
	15	137	0	20	27	27	89	171	12150	0	540	65	267	63	1358	33	76	403	122	1062	142
	16	474	43	37	17	80	1164	779	9579	140	420	616	547	117	2818	128	82	265	0	815	179
	18	605	0	102	59	105	117	338	15389	8	0	8	335	218	4401	270	1076	17	0	64	735
	19	411	0	54	56	24	793	434	10833	0	55	78	0	95	3766	137	117	13	85	61	0
	20	267	31	37	22	45	61	219	9002	25	179	66	60	53	2429	160	116	8	25	168	408
	21	356	55	57	206	42	1321	513	431	2	341	47	160	146	937	129	97	262	2	572	2242
	23	238	0	35	21	0	49	184	13470	0	19	0	1348	103	1807	64	78	19	0	74	464
	103	1158	231	113	83	66	454	336	385	0	0	4	1849	413	489	264	138	54	0	180	2148
Group 2	3	410	747	59	44	76	309	286	174	170	8351	278	313	792	17593	157	58	8279	286	13939	654
	6	315	0	51	59	131	141	169	12387	56	154	2	158	132	432	123	96	23	0	179	1149
	7	184	0	43	41	22	192	213	6191	0	34	77	44	8	272	129	47	0	141	84	252
	10	186	0	29	25	95	348	298	5578	267	1088	1114	820	139	475	68	117	831	6	1912	314
	17	206	0	28	34	14	318	242	207	6	7	7	430	47	597	90	46	6	0	26	163
	22	130	0	18	27	94	64	153	5896	154	438	606	515	278	155	137	60	161	0	662	125
	24	121	0	19	22	10	120	147	12313	1	0	0	251	24	208	90	42	31	0	13	0
	25	358	44	42	56	42	706	709	14773	100	40	53	0	39	2176	84	89	6	66	42	298
	26	331	0	63	66	134	388	556	12557	191	100	379	366	130	974	178	185	69	32	433	0
	27	163	0	45	40	48	275	242	11102	11	288	43	356	17	490	129	99	236	28	519	0
	28	128	0	16	25	58	52	126	9524	140	308	345	598	60	438	105	86	146	0	546	306
	29	324	0	69	56	44	273	277	17657	23	57	0	955	138	655	275	113	3	13	53	1178
Group 3	14	407	80	100	233	136	115	260	20863	49	129	111	672	243	4381	188	2317	0	0	262	343
	30	270	0	55	65	915	193	321	126	56	253	0	813	1522	21862	109	67	62	0	66	21
	31	387	0	74	61	27	107	237	109	114	147	115	979	207	358	201	86	9	37	122	230
	32	516	1349	66	12125	48668	304	227	184	190	69	231	605	196	355	140	63	42	273	318	1347
	34	482	0	36	14	12	224	273	16082	327	241	506	1235	132	5869	126	1079	127	0	564	354
	35	8425	5470	7937	3403	36	151	236	66	29	0	20	454	30	334	1170	43	11	0	22	0
	37	12026	548	8608	4297	16471	418	452	245	15	0	36	67	11	973	328	60	0	65	1074	281
	43	468	0	58	14232	15215	107	237	95	108	0	90	497	245	210	338	70	41	1225	0	424
	44	234	62	4	8	45	155	148	3761	190	93	145	414	55	470	78	48	19	0	54824	199
	46	260	0	35	1122	1171	59	107	56	300	198	42	1034	902	16932	85	30	87	0	0	444
	47	7448	5989	5865	2069	5	201	271	121	242	129	291	1454	72	389	993	46	66	0	370	45
	102	192	0	32	7333	522	82	171	75	12	0	0	1362	100	204	143	49	0	0	15	0
Group 4	33	205	0	2	66	0	169	129	8806	18	235	4	353	0	549	52	69	151	0	407	168
	36	369	0	47	31	56	190	225	133	75	2325	0	1199	1162	21563	176	64	3	0	145	342
	38	216	0	28	62	21	184	209	14622	0	75	187	834	90	411	122	68	38	16	101	244
	39	9798	1249	9809	3730	7056	125	139	109	0	248	43	430	0	270	472	25	117	0	1074	42
	40	6356	4693	5904	1570	261	314	453	175	2155	180	509	1412	120	903	1019	44	119	0	468	315
	41	278	0	41	18	63	232	159	11361	22	210	29	2027	171	2818	165	241	0	27	45	168
	42	178	0	14	12	77	151	170	10153	0	0	0	947	81	2625	68	65	0	0	1	270

Supplementary Figure 2. Binding of model lectins to *K. pneumoniae* isolates. Binding intensities to bacterial cells printed using suspensions with OD_{600nm} = 1 are shown. Data correspond to mean values of duplicate signal intensities. Median standard deviation was 25%. Abbreviations used for lectin names are defined in Supplementary Table 2, except that Ban and Jac state for BanLec and Jacalin, respectively.

	ID	HG1	HG3	HG4	HG4-N	HG4-C	HG7	HG8	HG8-N	HG8-C	HG9	HG9-N	HG9-C
Group 1	1	105	0	2522	2163	2043	204	2138	285	13359	23945	640	7496
	2	76	0	699	617	0	96	324	914	1436	6090	0	115
	4	65	1326	95	1043	604	84	2254	779	786	7380	0	3046
	5	199	350	3970	0	6028	138	7151	227	14315	27162	1498	24063
	8	127	884	1955	10216	2825	151	4470	118	6367	14473	670	3584
	9	93	0	1167	1028	2116	120	3749	540	9769	18388	836	8194
	11	52	2561	2258	2830	2946	118	1765	0	10404	23548	940	7122
	12	72	2323	2348	2239	5966	78	3635	7	13716	25222	290	7932
	13	1	2718	806	746	4360	159	2346	0	6777	13662	271	7581
	15	120	2706	1922	1889	4279	165	3874	495	9762	19719	338	5261
	16	97	0	2080	2069	3613	71	4257	130	9187	19085	1181	12627
	18	75	3316	2359	6413	8265	101	7413	874	11804	17162	4635	5428
	19	46	155	1411	1944	917	180	1218	54	10834	16560	1071	3446
	20	83	1224	1081	1401	1740	135	2053	0	9336	16111	244	1240
	21	99	152	49	574	0	155	485	120	1589	1320	61	1148
	23	60	0	3246	2310	10202	196	5626	0	13269	21442	1093	5896
	103	144	3058	304	1746	1377	202	1378	133	0	7638	222	1269
Group 2	3	100	1542	109	418	374	42	300	0	106	513	22	402
	6	41	259	1794	2786	1717	135	2144	58	11764	18720	1189	9060
	7	22	0	1472	1084	2028	39	1253	299	7545	14214	300	4829
	10	61	0	1974	1208	15946	154	2010	0	7978	18266	511	2881
	17	41	0	198	272	0	39	343	0	126	8101	66	2247
	22	89	911	1401	1155	4416	171	3986	49	9223	17203	1124	3228
	24	59	715	1879	4695	5785	193	4065	0	14294	19075	1740	3168
	25	79	623	2744	2707	4329	397	1794	49	10173	18726	1216	8089
	26	110	304	2901	4041	7170	590	2441	183	12117	16929	2638	14447
	27	114	620	1826	1395	7431	281	2116	0	9779	19285	1271	10314
	28	42	0	1738	1874	7660	77	3284	35	10692	16050	2287	11515
	29	74	2912	3327	2683	7125	218	5900	75	17607	28675	2769	13177
Group 3	14	58	7465	459	11140	13079	146	21334	254	37275	47575	23132	13258
	30	69	2289	191	445	242	120	22	0	75	680	44	2040
	31	184	6342	126	1103	741	296	35	0	199	0	129	2240
	32	283	9	444	1037	572	398	248	134	175	2107	95	3283
	34	86	1507	3503	11085	18876	440	6629	217	14920	22734	4109	20513
	35	43	0	800	691	0	105	338	0	125	673	155	1019
	37	103	0	0	638	543	49	216	0	306	602	69	1484
	43	816	0	110	724	532	484	8	0	119	5763	51	3819
	44	451	3066	50	234	151	132	9	1438	18	0	0	0
	46	493	109	64	314	93	1181	69	7	82	496	23	876
	47	123	2752	1174	605	1035	132	306	7	300	967	88	1978
	102	142	2645	0	563	0	118	279	0	0	347	28	846
Group 4	33	191	0	1386	860	4016	431	1872	0	7359	18302	793	7646
	36	51	2249	81	260	349	168	0	1168	28	331	62	2146
	38	185	800	1508	1323	2587	282	1215	415	6265	13174	1653	8535
	39	260	0	125	486	0	612	93	21	122	894	0	1644
	40	146	563	0	337	4835	107	169	0	85	811	103	1387
	41	46	0	2524	5761	26671	255	5052	200	16915	26985	5302	22302
	42	60	3423	61	2809	8727	468	4275	121	12675	21335	4285	19839

Supplementary Figure 3. Galectin binding to *K. pneumoniae* isolates. Binding intensities to bacterial cells printed using suspensions with OD_{600nm} = 1 are shown. Data correspond to mean values of duplicate signal intensities. Median standard deviation was 14%. HG, human galectin.

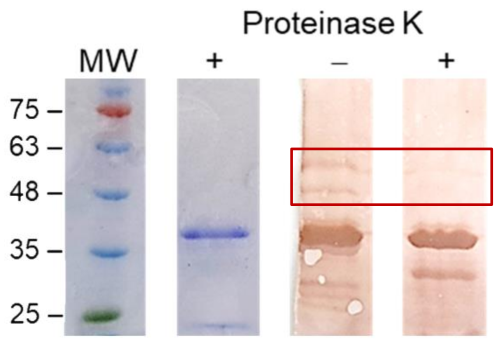

Supplementary Figure 4. Western blot analysis of the binding of galectin-9 to outer membranes (OM) of isolate 16 with (+) or without (–) pre-treatment with proteinase K. Electrophoretic and Western blot conditions were as in Figure 7 of the main manuscript, using 5 µL of OM stock preparation. From left to right: Molecular weight (in kDa) and electrophoretic mobility of markers; Coomassie Brilliant Blue staining; Western blot results obtained using non-treated and proteinase K-treated OM samples. Galectin-9-positive bands in the 63-48 kDa region are signaled by a red rectangle.

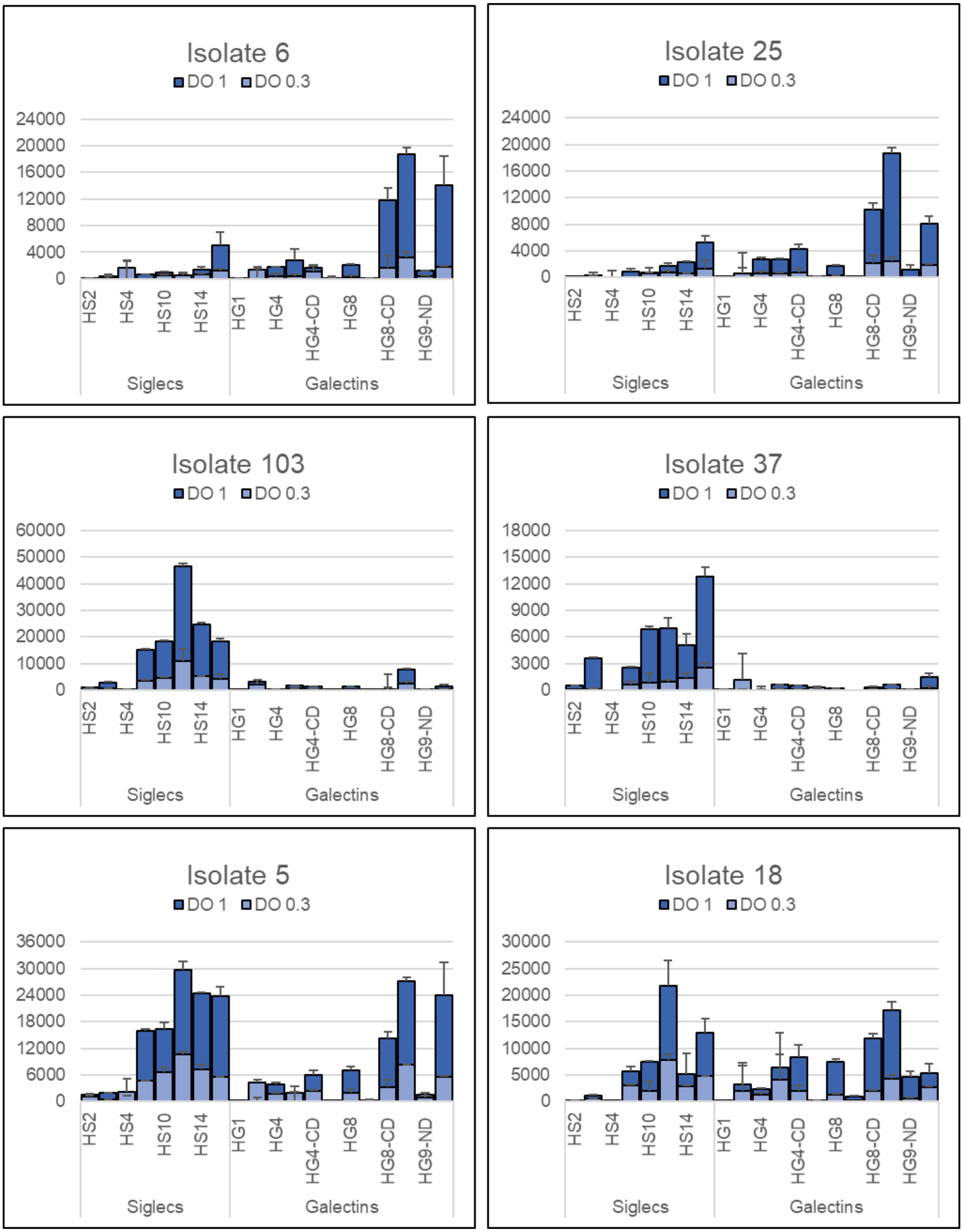

Supplementary Figure 5. Comparison of Siglec and galectin binding to selected *K. pneumoniae* isolates. Fluorescence intensities to bacterial cells printed using suspensions with OD_{600nm} = 1 (dark blue) and 0.3 (light blue) are shown. Data correspond to mean values of duplicate signal intensities and error bars indicate the standard deviation to the mean.

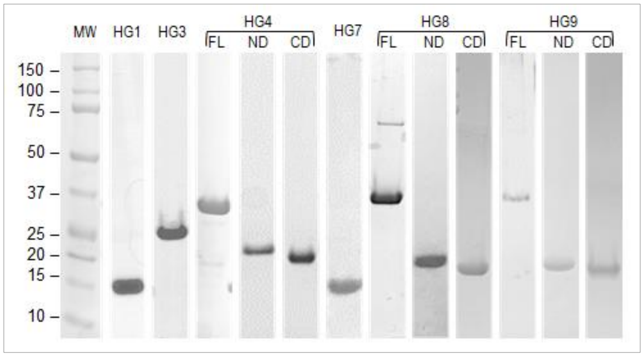

Supplementary Figure 6. Electrophoretic analysis of recombinant human galectins (HG). SDS-PAGE was performed using 4-12% gradient acrylamide gels (Invitrogen), under reducing conditions. Protein bands were visualized by Coomassie Brilliant Blue staining. The molecular weight (in kDa) and electrophoretic mobility of markers is indicated on the left. FL, full-length protein; ND, N-terminal carbohydrate-recognition domain; CD, C-terminal carbohydrate-recognition domain.

Supplementary Table 1. Sialic acid content of *K. pneumoniae* clinical isolates. ND, not determined

Group	Isolate ID	Capsular Serotype	MLST	μmol/10^9 bacterial cells
1	1	K1	23	0.010
	2	K1	23	0.009
	4	K1	23	0.008
	5	K1	23	0.009
	8	K1	23	0.010
	9	K1	23	0.010
	11	K1	23	ND
	12	K1	23	0.008
	13	K1	23	0.010
	15	K1	23	ND
	16	K1	23	ND
	18	K1	23	ND
	19	K1	23	0.010
	20	K1	23	0.008
	21	K1	23	0.009
	23	K1	23	0.009
	103	K1	81	ND
2	3	K2	25	0.008
	6	noK1/K2	1013	0.008
	7	K2	380	ND
	10	K2	380	ND
	17	K2	86	0.009
	22	K2	380	0.008
	24	K2	86	ND
	25	K2	86	0.009
	26	K2	65	0.008
	27	noK1/K2	1013	0.007
	28	noK1/K2	1013	0.007
	29	K2	493	0.008
3	14	K30	416	ND
	30	noK1/K2	1035	0.010
	31	noK1/K2	895	0.014
	32	noK1/K2	895	0.011
	34	K35	460	0.009
	35	noK1/K2	tlv719	ND
	37	noK1/K2	622	ND
	43	K24	45	0.010
	44	K15/K17/K50/K51/K52	37	0.011
	46	K26	321	0.012
	47	K26	17	0.009
	102	noK1/K2	152	0.012
4	33	K22.37	35	0.010
	36	K26	17	0.009
	38	K22.37	35	0.009
	39	K54	214	0.024
	40	K31	104	0.010
	41	noK1/K2	711	0.010
	42	noK1/K2	465	0.009

Supplementary Table 2. Model lectins used in microarray binding assays.

Lectin	Abbrv.	Nominal specificity	Binding preferences	Buffer[a]	Source[b]
Galanthus nivalis agglutinin	GNA	Man	Manα(1,3)Man	PBS	EY Labs
Hippeastrum hybrid lectin	HHL	Man	Manα(1,3/6)Man	PBS	Vector Labs
Narcissus Pseudonarcissus lectin	NPL	Man	Manα(1,6)Man; internal Man	PBS	Vector Labs
Musa paradisiaca lectin	BanLec	Man/Glc	α-MeMan > α-Man > α-Glc	PBS	Vector Labs
Concanavalin A	ConA	Man/Glc	α-Man as part of a “core” trimannoside. α-MeMan > α-Man > α-Glc > α-GlcNAc	TBS-Ca	Sigma
Pisum sativum agglutinin	PSA	Man/Glc	α-MeMan, α-Man, α-Glc; substitution at position 6 prevents binding	TBS-Ca	EY Labs
Vicia ervilia agglutinin	VEA	Man/Glc	Man and Manα(1,1)αMan; Glc, fructose, GlcNAc	PBS-Ca	BioWorld
Datura stramonium lectin	DSL	GlcNAc	β(1,4)-linked GlcNAc oligomers: Chitotriose > chitobiose > GlcNAc. Also LacNAc and LacNAc oligomers	PBS	Vector Labs
Lycopersicon esculentum agglutinin	LEA	GlcNAc	β(1,4)-linked GlcNAc oligomers, able to accommodate up to 4 GlcNAc units	PBS	Sigma
Wheat germ agglutinin	WGA	GlcNAc	$GlcNAc_3$, $GlcNAc_2$, GlcNAc; also Neu5Ac	PBS	Sigma
Agaricus bisporus agglutinin	ABA	Gal	Galβ(1,3)GalNAc (T-antigen), sialylated or non-sialylated; also binds GlcNAc at a different site	PBS	Vector Labs
Erythrina cristagalli lectin	ECL	Gal	LacNAc, Lac, GalNAc, Gal, non-sialylated	PBS	Vector Labs
Griffonia simplicifolia lectin I isolectin B4	GSL-IB4	Gal	Galβ(1,3)GlcNAc; Galα(1,6)Glcα(1,2)Fru	PBS-Ca	Sigma
Artocarpus integrifolia lectin	Jacalin	Gal	Galβ(1,3)GalNAc (T-antigen), sialylated or non-sialylated ; also Galα(1,6)Glc	PBS-Ca	Vector Labs
Maackia amurensis lectin I	MAL-I	Gal	LacNAc, Lac	PBS	Vector Labs
Peanut agglutinin	PNA	Gal	Galβ(1,3)GalNAc, Lac	PBS	Vector Labs

Ricinus communis agglutinin	RCA	Gal	Terminal β-Gal; Lac >> Galβ(1,3)Glc	PBS	Vector Labs
Ricinus communis lectin II	RICIN	Gal/GalNAc	Terminal β-Gal; Lac, Galβ(1,3)Glc, GalNAc	PBS	Purified in-house[d]
Viscum album agglutinin	VAA	Gal	Terminal β- and α-Gal	PBS	EY Labs
Dolichos biflorus agglutinin	DBA	GalNAc	Terminal non-reducing α-GalNAc	PBS	Vector Labs
Griffonia simplicifolia lectin I	GSL-I	GalNAc/Gal	Terminal α-GalNAc/Gal	PBS-Ca	Sigma
Helix pomatia agglutinin	HPA	GalNAc	α-GalNAc, β-GalNAc, α-Gal	TBS-Ca	Sigma
Iris hybrid agglutinin	IRA	GalNAc	GalNAcα(1,3)Gal; but not GalNAcα(1,3)GalNAc or GalNAcα(1,3)[Fucα(1,2)]Gal	PBS	GlycoMatrix
Maclura pomifera agglutinin	MPA	GalNAc/Gal	Internal GalNAc residues, Galβ(1,3)GalNAc, GalNAc/Gal; also Galα(1,6)Glc	PBS	EY Labs
Soybean agglutinin	SBA	GalNAc/Gal	Terminal α/β-linked GalNAc or Gal	PBS-Ca	EY Labs
Salvia sclarea agglutinin	SSA	GalNAc	αGalNAc	PBS	EY Labs
Vicia villosa lectin	VVL	GalNAc	Terminal α- or β-linked GalNAc; also Galα(1,3)GalNAc	PBS	Vector Labs
Wistera floribunda lectin	WFL	GalNAc	GalNAcα/β(1,3/6)Gal; also Lac	PBS	Vector Labs
Aleuria aurantia lectin	AAL	Fuc	Fuc α(1,3/6)-linked to GlcNAc or LacNAc related structures	PBS	Vector Labs
Ulex europaeus agglutinin I	UEA-I	Fuc	α(1,2)-linked Fuc; Ara	PBS	EY Labs
Arum maculatum agglutinin	AMA	Neu5Ac	Neu5Ac, LacNAc	PBS	EY Labs
Maackia amurensis lectin II	MAL-II	Neu5Ac	Neu5Acα(2,3)Galβ(1,3)GalNAc	PBS	Vector Labs
Polyporus Squamosus lectin	PSqL	Neu5Ac	Neu5Acα(2,6)Galβ(1,4)GlcNAc	PBS	EY Labs
Sambucus nigra lectin	SNL	Neu5Ac	Neu5Acα(2,6)Gal/GalNAc; also GalNAc and Lac	PBS	Vector Labs

[a]PBS: 5 mM sodium phosphate, pH 7.2, 0.2 M NaCl; PBS-Ca: PBS containing 0.5 mM $CaCl_2$; TBS-Ca: 10 mM Tris pH 8, 150 mM NaCl, 5 mM $CaCl_2$. [b]All commercial lectins were purchased in the form of biotin-conjugates. [c]Purified by affinity chromatography and biotinylated using standard procedures. LacNAc: N-acetyl-lactosamine (Galβ(1,4)GlcNAc); Lac: lactose (Galβ(1,4)Glc); α-MeMan: α-Methyl-D-mannopyranoside.
